# The Role of Dietary Fibers in the Management of Type 2 Diabetes: A Synthesis of Current Evidence and Clinical Implications

**DOI:** 10.3390/nu18040691

**Published:** 2026-02-21

**Authors:** Finta Hajnal, Ruța Florina, Avram Călin, Toncean Florentina Simona, Georgescu Mihai

**Affiliations:** 1 Department of Public Health and Health Management, George Emil Palade University of Medicine, Pharmacy, Science and Technology of Targu Mures, 540139 Targu Mures, Romania; hajnal.finta@umfst.ro; 2 Department of Community Nutrition and Food Safety, George Emil Palade University of Medicine, Pharmacy, Science and Technology of Targu Mures, 540139 Targu Mures, Romania; 3 Department of Medical Informatics and Biostatistics, George Emil Palade University of Medicine, Pharmacy, Science and Technology of Targu Mures, 540139 Targu Mures, Romania; 4 Department of Management, Dr. Eugen Nicoară Municipal Hospital, 545300 Reghin, Romania; s.boariu@yahoo.com; 5 Obstetrics Gynecology 1st Department, Braila County Emergency Hospital, 810325 Braila, Romania; ion_mihai_georgescu@yahoo.com

**Keywords:** type 2 diabetes, soluble dietary fibers, gut microbiota, short-chain fatty acids (SCFAs), clinical nutrition

## Abstract

Type 2 diabetes (T2D) represents a major public health challenge, being associated with significant metabolic and cardiovascular complications. Evidence-based nutritional interventions are essential for the prevention and management of the disease. Dietary fibers, particularly soluble fibers such as psyllium, β-glucan, inulin, and fermentable fiber blends, have demonstrated beneficial effects on glycemia, glycated hemoglobin (HbA1c), lipid profile, body weight, and medication requirements. This narrative review synthesizes the results of recent clinical trials and meta-analyses, highlighting the underlying physiological mechanisms, including colonic fermentation and short-chain fatty acid (SCFA) production, as well as the impact on gut microbiota composition. The findings support the integration of soluble fibers into a personalized dietary plan as part of a multidimensional strategy for T2D management. Further long-term randomized studies are warranted to standardize doses and assess the metabolic and microbiota-mediated effects of dietary fibers.

## 1. Introduction

Type 2 diabetes mellitus (T2DM) is one of the most prevalent chronic metabolic disorders worldwide. It is characterized by disturbances in carbohydrate, lipid, and protein metabolism resulting from insulin resistance and progressive β-cell dysfunction, leading to chronic hyperglycemia [1,2]. T2DM accounts for over 90% of all diabetes cases, and its continuously rising incidence represents a major global public health challenge [3,4]. The development and progression of T2DM are strongly associated with modifiable lifestyle-related risk factors, including overweight and obesity, physical inactivity, and unhealthy dietary patterns characterized by excess energy intake and poor nutritional quality, alongside genetic susceptibility and other environmental factors [5,6,7].

According to the International Diabetes Federation, approximately 537 million adults were living with diabetes worldwide in 2021. This number is projected to reach approximately 783 million by 2045 [7]. T2DM accounts for about 90% of all diabetes cases globally [7,8]. The increasing global burden of T2DM is multifactorial. It is strongly associated with modifiable risk factors such as overweight and obesity, physical inactivity, and unhealthy dietary patterns. These dietary patterns are characterized by excess energy intake, high consumption of saturated fats and refined carbohydrates, and low intake of dietary fiber. In addition, genetic susceptibility and environmental determinants interact with these factors [3,4,8,9].

In this context, nutritional interventions represent a fundamental component in the prevention and management of T2DM, alongside pharmacological therapy and regular physical activity [8,9]. Among dietary components influencing carbohydrate metabolism and glycemic control, dietary fiber, particularly soluble fiber, has been extensively studied for its metabolic benefits [9,10,11,12]. Soluble fibers are non-digestible polysaccharides that, in the presence of water, form viscous gels, thereby slowing gastric emptying and glucose absorption and modulating postprandial glycemic response [10,13]. Major dietary sources of soluble fiber include oats and barley (β-glucans), legumes (such as lentils, chickpeas, and beans), fruits (e.g., apples and citrus fruits, rich in pectin), and certain vegetables [10,14]. Numerous clinical and meta-analytic studies have demonstrated that increased soluble fiber intake is associated with reduced postprandial glycemia, improved insulin sensitivity, and better overall glycemic control in individuals with T2DM [11,15,16,17].

Research conducted over recent decades has clarified several mechanisms through which soluble dietary fibers contribute to glycemic regulation. Owing to their viscosity-forming properties, soluble fibers delay gastric emptying, slow intestinal glucose absorption, and attenuate postprandial glycemic excursions [10,13]. Fermentable soluble fibers resist digestion in the small intestine and are fermented by colonic bacteria, producing short-chain fatty acids (SCFAs) such as acetate, propionate, and butyrate [18,19,20]. SCFAs have multiple metabolic effects, enhancing gut barrier integrity, modulating inflammation, stimulating incretin secretion (e.g., GLP-1 and PYY), and improving insulin sensitivity, which together support glucose homeostasis [18,19,20]. Clinical and meta-analytic evidence further supports the role of soluble fiber intake in improving glycemic control in individuals with T2DM [11,17]. Together, these mechanisms underscore the relevance of soluble dietary fiber as a complementary nutritional strategy in T2DM management.

Major international organizations emphasize the importance of dietary fiber in preventing and managing T2DM. The American Diabetes Association (ADA) and the European Association for the Study of Diabetes (EASD) recommend healthy dietary patterns with adequate fiber intake, highlighting the benefits of fiber-rich foods for glycemic control and cardiometabolic health [9,21]. In line with this, the World Health Organization advises a minimum intake of 25 g/day of naturally occurring dietary fiber from foods for adults, with age-specific lower recommendations for children [22]. Similarly, the European Food Safety Authority (EFSA) recommends 25 g/day of dietary fiber for adults to support normal bowel function [13]. Large-scale meta-analyses further show that higher dietary fiber intake is associated with reduced incidence of cardiovascular disease, T2DM diabetes, and overall mortality [14].

Nutritional management of T2DM primarily aims to normalize body weight, optimize glycemic control, and improve the lipid profile [9]. Current guidelines recommend the consumption of low–glycemic index foods, high in dietary fiber, reduction in simple sugars, and limitation of saturated fat intake [8]. In particular, soluble dietary fibers have attracted considerable interest due to their beneficial effects on glycemia and lipid metabolism.

Adequate intake of soluble fiber has been associated with reductions in glycated hemoglobin (HbA1c), a key marker of long-term glycemic control [23]. It is also linked to increased satiety and reduced energy intake, contributing to weight loss, an essential factor in the prevention and management of T2DM [24]. Through colonic fermentation, soluble fibers produce short-chain fatty acids (SCFAs), such as acetate, propionate, and butyrate, improve insulin sensitivity, and influence the secretion of intestinal hormones involved in appetite regulation and energy metabolism [18].

In addition, high-fiber diets contribute to reduced cholesterol absorption and lower LDL-cholesterol levels, thereby decreasing the cardiovascular risk associated with T2DM [25,26]. They also reduced the risk of other metabolic disorders, including metabolic syndrome and non-alcoholic fatty liver disease [27]. Dysbiosis of the gut microbiota, genetic factors, and unhealthy dietary patterns further contribute to the pathogenesis of T2DM [28,29,30].

Soluble fibers exert beneficial metabolic effects through complex mechanisms that include delayed carbohydrate absorption, modulation of enteroendocrine hormone secretion, and reduction in glycemic variability [10,11]. Their fermentation leads to the formation of metabolites with important roles in reducing inflammation and improving insulin sensitivity [19,20]. Consequently, higher soluble fiber intake is associated with a lower dietary glycemic index and improved glycemic control, reducing the risk of chronic complications of T2DM [17].

Clinical studies have shown that the inclusion of soluble fiber in the diets of individuals with T2DM can reduce HbA1c by approximately 0.3–0.6% when total fiber intake exceeds 25 g/day, with at least 7–10 g/day derived from soluble fiber [31]. The meta-analysis conducted by Reynolds et al. (2019) highlighted the association between higher fiber intake and reduced T2DM, related mortality, lower HbA1c, fasting plasma glucose (FPG), and insulin resistance [12]. The effectiveness of soluble fiber depends on its type, dose, and dietary context, with beta-glucans from oats and barley demonstrating significant effects at doses ≥ 3 g/day [14].

Given the increasing prevalence of T2DM and its major socioeconomic impact [7,8], evidence-based nutritional interventions represent an essential strategy for disease prevention and management [32]. Soluble fibers such as pectin, beta-glucans, and inulin have demonstrated favorable effects on glycemic control and lipid profiles [15,33]. Recommendations from the European Food Safety Authority (EFSA) and the American Diabetes Association support adequate fiber intake as an integral component of T2DM management [1,34,35].

However, the scientific literature reports variable outcomes, influenced by study heterogeneity, types of fiber used, dosages, and characteristics of the populations investigated [35], highlighting the need for an integrated analysis of the existing evidence.

In this context, the aim of the present study was to analyze and synthesize current scientific evidence regarding the role of dietary fiber in regulating carbohydrate metabolism in individuals with T2DM and disorders of glucose homeostasis. Specifically, the study sought to:

1. Evaluate the effects of soluble and insoluble dietary fibers on glycemic control by analyzing changes in FPG, glycated hemoglobin (HbA1c), postprandial glycemic response, and insulin sensitivity;
2. Investigate the role of colonic fiber fermentation and short-chain fatty acids (SCFAs) as metabolic mechanisms involved in the improvement of insulin resistance and systemic inflammation;
3. Analyze the interaction between fiber intake and the gut microbiota, with emphasis on changes in bacterial composition, SCFA production, and their impact on the microbiota–inflammation–carbohydrate metabolism axis;
4. Identify mechanistic and clinical differences between soluble and insoluble fibers, as well as their implications for nutritional strategies in the prevention and management of T2DM.

By integrating data from randomized clinical trials, crossover studies, and meta-analyses, this work aims to provide a comprehensive perspective on the role of dietary fiber as an adjunct nutritional intervention in the management of metabolic dysfunctions.

## 2. Materials and Methods

This scoping review was conducted based on a structured literature search for relevant scientific publications. The literature included in this review was selected from peer-reviewed journals with high scientific impact, such as The American Journal of Clinical Nutrition, Clinical Nutrition, Diabetes Care, Diabetologia, The Journal of Nutrition, Nutrition Reviews, Gut, The Lancet, and JAMA, last search: 10 January 2026, ensuring methodological rigor and relevance to nutritional and metabolic research. Search terms were combined using Boolean operators (AND to link related concepts, OR for synonyms, NOT to exclude irrelevancies).

For the formulation of the research question and the structuring of the selection criteria, the PICO model (Population, Intervention, Comparison, Outcome), widely recognized in clinical research methodology and systematic reviews, was applied. This framework allowed for a clear definition of the key elements of the analysis:

- Population (P): adult patients diagnosed with T2DM;
- Intervention (I): increased intake of soluble dietary fiber (diet or supplementation);
- Comparison (C): standard diet or low fiber intake;
- Outcome (O): changes in clinically relevant parameters such as blood glucose, HbA1c, HOMA-IR, and lipid profile.

Using the PICO framework, the research question was formulated as follows: “In patients with T2DM, does an increased intake of soluble dietary fiber, compared with a standard diet, influence glycemic control and lipid profile?” This approach enabled a rigorous selection of studies and a focused analysis aligned with clinically relevant objectives.

### 2.1. Selection and Data Charting

Records were deduplicated (manual/Excel). Two reviewers independently screened titles/abstracts and full texts, resolving disagreements by consensus. Data items (fiber type, dose in g/day, duration, outcomes) were extracted using a standardized Excel form.

**Figure 1:**
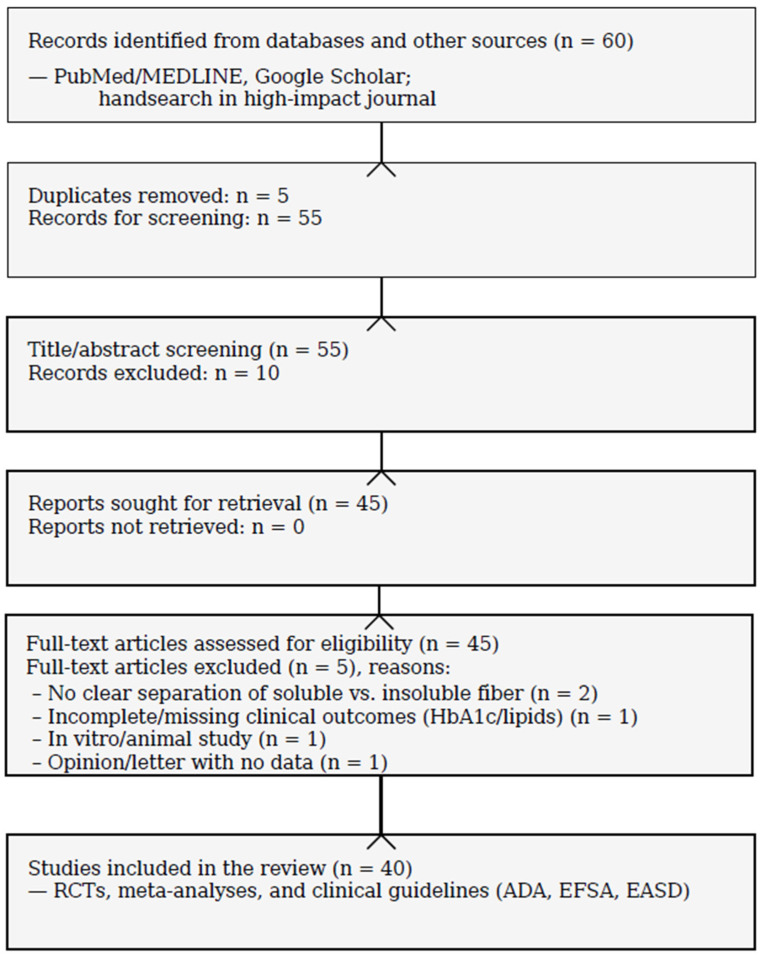
PRISMA 2020—Scoping Review: T2DM & soluble fiber.

Synthesis. Given heterogeneity across interventions and outcomes, we performed a structured narrative synthesis by themes (glycemic control, insulin resistance indices, lipid profile, tolerability) and cross-referenced international guidelines (ADA, EFSA, EASD). A PRISMA 2020 flow diagram (scoping) summarizes study selection (Figure 1). We did not conduct a formal risk-of-bias assessment, consistent with a scoping objective.

### 2.2. Inclusion Criteria

To ensure the relevance and quality of the analyzed studies, rigorous inclusion criteria were established. Publications written in English and published in recognized scientific journals within the last 10–15 years were included, except for seminal articles considered essential for the theoretical background. We included randomized controlled clinical trials, meta-analyses, and international guidelines; only studies reporting clinically relevant outcomes aligned with the objectives (e.g., blood glucose, HbA1c, lipid profile) were retained.

### 2.3. Exclusion Criteria

Studies conducted on animals/in vitro models were excluded, as were publications lacking experimental data, including opinion papers and letters to the editor. Articles that did not clearly distinguish between soluble and insoluble dietary fibers were also excluded, as this distinction was essential to the objectives of the review. Additionally, studies with unclear design or incomplete data were omitted to maintain a high level of methodological rigor.

### 2.4. Data Extraction and Analysis

The analysis process was conducted in several stages to ensure consistency and robustness of interpretation. More than 60 sources were initially reviewed, of which 40 were selected based on relevance and methodological quality. The initial stage involved critical reading of the articles, focusing on study design, applied methodology, statistical outcomes, and characteristics of the investigated populations. Subsequently, data extracted from the selected studies were synthesized into summary tables, including information on fiber types, dosages, intervention duration, and observed metabolic effects. The final synthesis followed a thematic approach, structuring the content according to the underlying physiological mechanisms and the impact of dietary fiber on glycemic control, lipid profile, body weight, and digestive tolerance. In addition, the results were compared against official recommendations issued by international guidelines, such as those from the American Diabetes Association (ADA), the European Food Safety Authority (EFSA), and the European Association for the Study of Diabetes (EASD), in order to assess the consistency between the analyzed data and current standards.

### 2.5. Limitations

This scoping review has several limitations that merit explicit mention. First, doses and dosing schedules varied substantially. Reported exposure ranged from modest daily intakes to higher, clinically oriented amounts, with further variation in formulation (isolated supplement vs. food matrix), timing relative to meals, and adherence. Many studies relied on self-report or capsule counts, whereas objective compliance markers were uncommon; consequently, true exposure may be uncertain in some reports.

Second, the study populations were not uniform. Participants differed by baseline glycemic status (T2DM with varying HbA1c targets, prediabetes, mixed metabolic profiles), age, comorbidities, background diet, and concomitant antihyperglycemic therapy (e.g., metformin, insulin, other agents). These features can modify observed effects (for example, larger HbA1c reductions at higher baselines), yet stratification or statistical adjustment was not consistently applied, which limits comparability and the generalizability of findings to specific subgroups.

Third, intervention length ranged from acute or short-term exposure (days to weeks) to longer trials (several months). Outcomes such as HbA1c and, often, lipids evolve over longer time windows; thus, short trials may understate clinically relevant changes, whereas longer trials are more vulnerable to co-intervention drift (e.g., medication titration, weight change, shifts in background diet) that may complicate causal interpretation.

Fourth, we noted inconsistencies in endpoint definitions and statistical reporting (e.g., fasting plasma vs. blood glucose; handling of missing data; uneven reporting of variance and uncertainty), which complicate numerical synthesis across studies. Several trials also appeared underpowered for secondary outcomes (e.g., insulin-resistance indices, lipids), and selective endpoint reporting cannot be ruled out.

Fifth, there were methodological differences in how gut microbiota and short-chain fatty acids (SCFAs) were measured. Microbiome profiling approaches (e.g., 16S rRNA vs. shotgun metagenomics), sequencing depth, bioinformatic pipelines, and normalization strategies varied, affecting taxonomic resolution and functional inference. SCFAs were assayed in stool and/or plasma using different analytical platforms (GC-FID, GC-MS, LC-MS), sampling schedules (fasted vs. postprandial; single vs. repeated sampling), and pre-analytical procedures, all of which can shift absolute concentrations and relative patterns. Non-uniform units and reporting formats further limit cross-study comparison and weaken links between microbiota/SCFA changes and clinical endpoints (HbA1c, HOMA-IR, lipids).

Finally, as a scoping review, our methods emphasized breadth and clinical interpretability rather than exhaustive retrieval or formal meta-analysis. Although we used explicit inclusion/exclusion criteria and a structured search, some eligible studies may lie outside our sources or time window. Taken together, these constraints highlight the need for fiber-specific, dose-ranging, adequately powered trials with standardized protocols for microbiota/SCFA sampling and analysis, pre-specified clinical endpoints, and transparent tracking of adherence and medication changes, to clarify causality and improve translational relevance.

## 3. Results

### 3.1. Effects of Soluble Dietary Fibers on Glucose Metabolism

The present research included randomized controlled clinical trials and meta-analyses that evaluated the effects of soluble dietary fibers on glycemic control in individuals with T2DM and prediabetes. The analyzed studies involved sample sizes ranging from 40 to more than 2600 participants, while the meta-analyses aggregated data from up to 46 randomized controlled trials (Table 1). The effects of soluble fibers on glycemic control and other metabolic parameters were assessed using standardized indicators such as fasting plasma/blood glucose (FPG/FBG), glycated hemoglobin (HbA1c), the HOMA-IR index, lipid profile, and, in some cases, insulin requirements.

Early randomized controlled trials demonstrated favorable effects of high-fiber foods on glycemic control. Jenkins et al. (2012) reported significant reductions in HbA1c and postprandial glucose following a dietary intervention based on increased legume consumption [36]. Similar findings were reported by Dall’Alba et al. (2013), who observed reductions in HbA1c and fasting glucose, as well as improvements in the metabolic profile, following supplementation with partially hydrolyzed guar gum [37].

Psyllium supplementation has been evaluated in several randomized controlled trials. Feinglos et al. (2013) demonstrated that psyllium administration for 8 weeks led to a significant improvement in glycemic control, including reductions in fasting and postprandial glucose, without the occurrence of major adverse effects [38]. These findings were subsequently confirmed by Abutair et al. (2016), who reported significant decreases in fasting glucose, HbA1c, insulin levels, and the HOMA-IR index following an 8-week psyllium intervention [39].

Interventions using various types of soluble fibers have also demonstrated beneficial effects on glucose metabolism. Thompson et al. (2017) observed significant improvements in fasting glucose, 2 h postprandial glucose, and insulin resistance, assessed by HOMA-IR, after a 17-week intervention [40]. At the level of evidence synthesis, the meta-analysis conducted by Wang et al. (2019) showed that inulin-type fructans were associated with significant reductions in fasting glucose, HbA1c, and insulin resistance, with dose-dependent effects and more pronounced benefits at doses ≥ 10 g/day [41].

Over the long term, data synthesized by Reynolds et al. (2020) suggest that higher soluble fiber intake is associated with a reduced risk of T2DM, related complications, and improved glycemic control over a follow-up period of up to 10 years [42]. Similar results were reported by Mao et al. (2021), who observed significant reductions in HbA1c and fasting glucose following 8 weeks of soluble fiber supplementation [43].

The meta-analysis conducted by Xie et al. (2021), which included 29 randomized controlled trials, confirmed the beneficial effects of soluble fibers, reporting significant reductions in HbA1c, fasting glucose, insulin levels, and the HOMA-IR index [44]. Specifically, oat β-glucan was evaluated in a randomized controlled trial by Pino Villalón et al. (2021), which demonstrated significant improvements in fasting glucose and HbA1c, along with favorable effects on the lipid profile, after a 3-month intervention [45]. These findings are supported by the meta-analysis by Zurbau et al. (2021), which reported a significant reduction in postprandial glycemic and insulinemic responses in a dose- and viscosity-dependent manner [46].

Finally, recent meta-analyses have further strengthened the evidence regarding the efficacy of different types of soluble fibers. Juhász et al. (2022) identified galactomannans as the most effective fibers in reducing HbA1c and fasting glucose [47]. Lu et al. (2023) reported significant reductions in HbA1c, fasting glucose, and the HOMA-IR index following supplementation with 10–15 g/day of psyllium, guar, or β-glucan [48].

**Figure 2:**
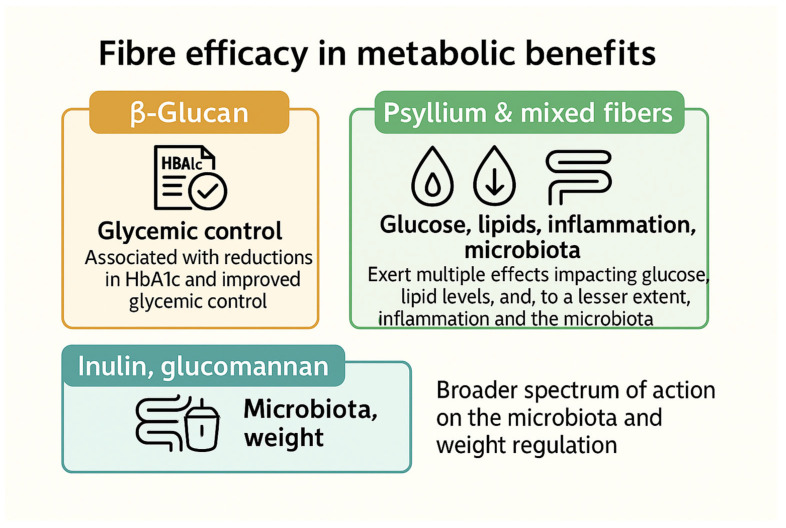
Multidimensional profile of the effects of soluble fibers on metabolic parameters.

β-Glucan is particularly associated with reductions in HbA1c and improved glycemic control, highlighting its efficacy in glycemic management. Psyllium and mixed fibers exert multiple effects, significantly impacting glucose levels, lipid profiles, and, to a lesser extent, inflammation and gut microbiota. Inulin and glucomannan show a broader spectrum of action on microbiota and weight regulation, suggesting additional indirect metabolic benefits (Figure 2).

### 3.2. Metabolic Effects Induced by the Fermentation of Dietary Fibers

To synthesize current scientific evidence on the relationship between dietary fiber intake, colonic production of short-chain fatty acids (SCFAs) through fermentation, and their effects on systemic inflammation and insulin resistance, randomized controlled trials and crossover studies published over the past approximately 10–15 years were identified and analyzed. Table 2 presents the main methodological characteristics of these studies, including the type of intervention applied, the metabolic parameters assessed, and the key reported outcomes. It highlights the impact of fermentable fibers and SCFAs on insulin sensitivity and glycemic response in overweight adults or those with metabolic disorders.

### 3.3. Role of Dietary Fibers in Modulating the Gut Microbiota

Table 3 summarizes key clinical trials and systematic reviews investigating the effects of dietary fibers on gut microbiota and glucose metabolism in patients with T2DM [56,57,58,59,60,61,62]. Randomized controlled trials show that high-fiber dietary interventions, including mixtures of fermentable fibers, inulin, galacto-oligosaccharides, and resistant dextrin, lead to favorable changes in microbiota composition, characterized by increased abundance of short-chain fatty acid–producing bacteria such as Bifidobacterium and Akkermansia. These changes are associated with improved glycemic control, reflected by reductions in HbA1c and increased GLP-1 secretion, as well as decreased systemic inflammation and metabolic endotoxemia. Additionally, the meta-analysis by Ojo et al. confirms, in a large sample, the beneficial effects of dietary fibers on gut microbiota and inflammatory markers, supporting their role as an adjunct nutritional strategy in the management of T2DM.

The analyzed clinical studies highlight clear differences between the effects of soluble and insoluble fibers on glucose metabolism in patients with diabetes or impaired glucose regulation. Soluble fibers, such as psyllium, β-glucans, and galactomannans, consistently demonstrate significant reductions in FPG, HbA1c, and postprandial glycemic responses [63,64]. These effects are attributed to the formation of viscous gels in the gastrointestinal tract, slowing glucose absorption, and stimulating incretin hormone secretion. In contrast, insoluble fibers, particularly those derived from cereals, appear to exert more modest and indirect metabolic effects, being associated with improved insulin sensitivity and reduced risk of progression to diabetes, especially in individuals with impaired baseline glucose [65,66]. These findings suggest that soluble and insoluble fibers act via distinct but complementary mechanisms, supporting the inclusion of both fiber types in nutritional strategies for diabetes management (Table 4).

## 4. Discussion

The present analysis consolidates evidence indicating that dietary fibers represent a major nutritional determinant of glycemic control and metabolic homeostasis in individuals with T2DM and glucose regulation disorders. In particular, the results indicate that soluble fibers exert consistent and clinically relevant effects on FPG, glycated hemoglobin, and insulin resistance, whereas insoluble fibers primarily contribute to improved insulin sensitivity and reduced risk of long-term metabolic progression. These observations align with the literature, which emphasizes the role of fibers not merely as passive dietary components but as active modulators of glucose metabolism and systemic inflammation [42,67]. Changes in microbiota composition are strongly correlated with intestinal dysbiosis and inflammatory markers, suggesting that bacterial balance is a determinant factor not only in T2DM but also in other conditions characterized by systemic inflammation. This context underscores the importance of microbiota modulation via fibers in metabolic management [68].

A central element of the findings is the consistency of soluble fiber effects on HbA1c, a key marker of long-term glycemic control. Although the observed reductions are moderate in magnitude, they are comparable to those achieved through standard dietary interventions and are considered clinically meaningful, particularly within multimodal therapeutic strategies. Similar evidence has been reported in recent meta-analyses, indicating that increased soluble fiber intake is associated with a significant reduction in the risk of T2DM and its cardiovascular complications [12,69].

Short-chain fatty acids (SCFAs) have been associated with enhanced insulin sensitivity, modulation of incretin hormone secretion (GLP-1, PYY), and reduction in low-grade inflammation [10,18,23,46]. These mechanisms indicate that the metabolic effects of soluble fibers extend beyond simple modulation of carbohydrate absorption, contributing to systemic metabolic regulation.

Viscous soluble fibers such as psyllium, oat β-glucans, and galactomannans appear to be particularly effective in improving glycemic control, especially at doses ≥ 10 g/day, as demonstrated in recent systematic reviews, network meta-analyses, and dose–response analyses in patients with T2DM [47,48,70]. These findings suggest that the metabolic benefits of fiber are influenced not only by total intake but also by physicochemical properties, including viscosity and fermentability.

A significant contribution of this analysis is the integration of fiber fermentation and SCFA production in improving insulin resistance. Studies using reference methods, such as the euglycemic–hyperinsulinemic clamp, demonstrate that resistant starch and fermentable fibers can enhance insulin sensitivity through propionate—and butyrate-dependent mechanisms. Propionate has been associated with inhibition of hepatic gluconeogenesis and stimulation of GLP-1 secretion, while butyrate plays a key role in maintaining intestinal barrier integrity and reducing metabolic inflammation [18,71].

In this context, modulation of the gut microbiota emerges as a key mechanism linking fiber intake to improved glucose metabolism. The analyzed studies report a consistent increase in SCFA-producing bacteria, such as Bifidobacterium and Akkermansia muciniphila, following interventions rich in fermentable fibers. These changes support the existence of a gut microbiota-inflammation-glucose metabolism axis [56,71].

Differences between soluble and insoluble fibers highlighted by the analyzed results reflect distinct but complementary mechanisms. Soluble fibers exert rapid and direct effects on glycemic control, whereas insoluble fibers, particularly those from whole grains, appear to influence glucose metabolism through indirect mechanisms, including improved insulin sensitivity and prevention of diabetes progression in at-risk individuals [72]. This complementarity suggests that optimal nutritional strategies should include both fiber types.

Plant-based dietary patterns provide insight into the clinical relevance of dietary fiber intake, particularly in the context of metabolic disorders. Vegan diets, characterized by the exclusion of animal-derived foods and a high consumption of whole grains, legumes, fruits, vegetables, nuts, and seeds, provide higher amounts of total, soluble, and fermentable fiber. Studies indicate that individuals following vegan dietary patterns have significantly higher fiber intakes compared to those consuming traditional omnivorous diets, along with more favorable anthropometric and cardiometabolic profiles [73,74].

In patients with T2DM, plant-based diets have been associated with improvements in glycemic control, insulin sensitivity, and lipid parameters. The review by Pollakova D et al. (2021) summarizes evidence supporting the role of vegan diets in both prevention and adjunctive treatment of T2DM, highlighting mechanisms that include increased fiber intake, modulation of gut microbiota composition, and attenuation of systemic inflammation [75]. Similarly, the systematic review and meta-analysis conducted by Lv M et al. (2025) reports significant reductions in HbA1c, body weight, and LDL-cholesterol among individuals with T2DM adhering to vegetarian or vegan dietary interventions [76].

These clinical findings are consistent with the data processed in the present study, namely that diets rich in viscous and fermentable fibers lead to an increased production of short-chain fatty acids (SCFAs) [77,78]. Recent evidence highlights the central role of gut microbiota-derived metabolites in metabolic regulation, with propionate and butyrate shown to improve insulin sensitivity, modulate hepatic glucose production, and reduce systemic inflammation [78]. SCFAs have also been implicated in the stimulation of incretin hormones, including GLP-1 and PYY, thereby contributing to improved glycemic control and appetite regulation [78].

Moreover, butyrate plays a critical role in maintaining intestinal barrier integrity and limiting metabolic endotoxemia, mechanisms closely linked to reduced low-grade inflammation and enhanced insulin responsiveness [76]. Plant-based dietary patterns, characterized by high intake of fermentable substrates, have been associated with increased abundance of SCFA-producing bacteria, including *Bifidobacterium* spp. and *Akkermansia muciniphila*. Human interventional data, such as those reported by Depommier et al. (2019), support the metabolic relevance of *Akkermansia muciniphila* in improving insulin sensitivity and inflammatory markers [79]. Additionally, recent analyses of vegetarian and vegan dietary patterns demonstrate consistent shifts toward a microbiota profile enriched in SCFA-producing taxa, further supporting the link between dietary fiber intake, microbial modulation, and glucose homeostasis [80].

However, interpretation of the results must consider the limitations of the available literature. Heterogeneity in interventions, the relatively short duration of many studies, and variability in individual responses largely determined by gut microbiota composition limit the generalizability of conclusions. Furthermore, direct causal relationships between microbiota changes, SCFA production, and improvement in metabolic parameters are difficult to demonstrate without in-depth functional analyses, such as metabolomics or isotopic tracing studies.

Current evidence supports the role of dietary fibers, particularly soluble and fermentable types, as essential nutritional tools in the prevention and management of T2DM. Their benefits are mediated through a combination of effects on carbohydrate absorption, colonic fermentation, SCFA production, and gut microbiota modulation. Long-term randomized clinical trials with standardized designs and multi-omics approaches are needed to clarify causal mechanisms and enable personalized nutritional recommendations based on individual metabolic and microbial profiles.

Nutritional education plays a central role in promoting adequate dietary fiber intake and fostering broader healthy eating behaviors that have consistently been associated with a lower risk of T2DM [81]. These behaviors include limiting fast-food consumption [82,83], reducing intake of free sugars and sugar-sweetened beverages [84], increasing consumption of unsaturated fats instead of saturated fats [85], and maintaining proper hydration through education on safe water sources [86]. They also involve maintaining a healthy body weight [87] and implementing structured lifestyle and self-management education strategies [88,89,90] for the prevention and management of T2DM and related metabolic disorders.

## 5. Conclusions

The results synthesized in this analysis consistently support the role of dietary fibers as important determinants of glycemic control and insulin sensitivity in individuals with T2DM and prediabetes. Available evidence shows that soluble fibers have direct and significant metabolic effects. These effects include reductions in fasting glucose, HbA1c, and postprandial glycemic responses. In contrast, insoluble fibers mainly improve insulin sensitivity and reduce the risk of long-term metabolic progression.

A central finding of this work is that the metabolic benefits of fibers are not explained solely by their physicochemical effects on carbohydrate digestion. They are also significantly mediated by colonic fermentation and the production of short-chain fatty acids (SCFAs), particularly butyrate and propionate. Increased SCFA concentrations are associated with enhanced insulin sensitivity, stimulation of incretin hormone secretion, reduced systemic inflammation, and maintenance of intestinal barrier integrity. These mechanisms support the existence of a functional gut–microbiota–glucose metabolism axis.

Furthermore, dietary interventions rich in fermentable fibers induce favorable modifications of the gut microbiota, characterized by increased abundance of SCFA-producing bacteria such as Bifidobacterium and Akkermansia, changes that are correlated with improved glycemic control and reduced metabolic endotoxemia. These observations emphasize the importance of fiber quality and type, not merely total intake.

However, the metabolic response to fiber supplementation exhibits considerable interindividual variability, influenced by baseline microbiota composition, metabolic status, dose, and duration of intervention. Limitations of the current literature include heterogeneity in study design, relatively small sample sizes, and lack of standardization in methods for assessing SCFAs and microbiota composition.

Through our study, we highlighted that the data from the specialized literature support the following observations: soluble fibers decrease fasting blood glucose, HbA1c and postprandial blood glucose, while insoluble fibers mainly increase insulin sensitivity and reduce long-term metabolic risk. In addition, the metabolic benefits of fermentable fiber include the increase in AGSC-producing bacteria, such as Bifidobacterium and Akkermansia, which in turn are associated with better glycemic control and reduced metabolic endotoxemia. At the same time, the effects of fiber supplementation are influenced by the initial microbiota, metabolic status, type and dose of fiber, and the duration of the intervention.

In conclusion, the integration of dietary fiber, especially soluble and fermentable fiber, can be considered an effective nutritional strategy for the prevention and management of T2DM. This conclusion would be even more solid if the data were complemented by randomized, long-term clinical trials with multi-omics analyses and personalized approaches, which would clarify the exact mechanisms and identify the subgroups of patients who would benefit most from these interventions.

## Figures and Tables

**Table 1:** Effects of Soluble Dietary Fibers on Glucose Metabolism in Patients with T2DM.

Reference	Study Type	Number of Participants	Intervention Duration	Type of Fiber	Reported Outcomes
Jenkins et al., 2012 [36]	Randomized clinical trial (RCT)	121	12 weeks	Legumes (190 g/day)	Reduction in HbA1c and postprandial glucose
Dall’Alba et al., 2013 [37]	RCT	44	6 weeks	Guar gum PHGG 10 g/day	Reduction in HbA1c, fasting glucose, and improvement in metabolic profile
Feinglos et al., 2013 [38]	RCT	30–40	8 weeks	Psyllium	Significant improvement in glycemic control: reductions in fasting and postprandial glucose; overall glycemic profile improved without major adverse effects
Abutair et al., 2016 [39]	RCT	40	8 weeks	Psyllium 10.5 g/day	Reduction in FBG, HbA1c, insulin, and HOMA-IR
Thompson et al., 2017 [40]	RCT	609	17 weeks	Various soluble fibers	Improvement in fasting and 2 h postprandial glucose levels, accompanied by a reduction in HOMA-IR.
Wang et al., 2019 [41]	Meta-analyze	1500 participants (from 33 randomized clinical trials, populations with prediabetes and type 2 diabetes)	2–24 weeks (depending on included studies)	Inulin-type fructans (inulin, oligofructose, FOS—fermentable soluble fibers)	Significant improvement in glycemic control: reduction in fasting glucose, HbA1c, and insulin resistance; dose-dependent effects (greater benefits at ≥10 g/day); moderate–high quality of evidence (GRADE)
Reynolds et al., 2020 [42]	Meta-analyze	1789	10 years	15–35 g various soluble fibers	Higher soluble fiber intake associated with reduced risk of T2D complications and better glycemic control
Mao et al., 2021 [43]	RCT	911	8 weeks	Various soluble fibers, 10 g/day	Significant reductions in HbA1c and fasting glucose
Xie et al., 2021 [44]	Meta-analyze	1517 (29 RCT)	variable	Psyllium, β glucan, etc.	Reductions in HbA1c (−0.63%), FBG, insulin, HOMA-IR
Pino Villalon et al., 2021 [45]	RCT	50–60	3 months	Oat β-glucan (a soluble, viscous fiber)	Significant reduction in fasting glucose and HbA1c; improvement in overall glycemic control; beneficial effects on the lipid profile; supplementation was well tolerated, with no significant adverse effects reported.
Zurbau et al., 2021 [46]	Meta-analyze	>600	variable	Oat β glucan (soluble, viscous fiber)	Significant reduction in postprandial glucose and insulin responses; reduced area under the curve (AUC) for glucose and insulin
Juhász et al., 2022 [47]	Meta-analyze	2685 (46 RCT)	variable	Galactomannans, psyllium, β-glucan	Galactomannans are the most effective in reducing HbA1c and FBG.
Lu et al., 2023 [48]	Meta-analyze	2000	12 weeks (average)	10–15 g psyllium, guar, β glucan	Reduction in HbA1c, FBG, and HOMA-IR; improved glycemic control

**Table 2:** SCFAs—Human Randomized Controlled Trials and Metabolic Data.

Reference	Study Type	Intervention	Measured Parameters	Key Results
Johnston et al. (2010) [49]	RCT, placebo-controlled	Resistant starch 40 g/day, 12 weeks	Insulin sensitivity via euglycemic clamp	Improved insulin sensitivity in metabolic syndrome
Bodinham et al. (2012) [50]	RCT, crossover	HAM-RS2 vs. placebo la T2DM	Postprandial glucose and insulin	Reduced postprandial glycemic response; variable effects on sensitivity
Rahat-Rozenbloom et al. (2017) [51]	RCT, crossover	Inulin 24 g + RS 28 g, acute intervention	Serum SCFA and insulin response	Inulin increased serum SCFA; RS reduced postprandial insulin
Chambers et al. (2019) [52]	RCT, crossover, double-blind	Inulin-propionate ester vs. inulin vs. cellulose, 42 days	HOMA-IR, serum/fecal SCFA, inflammatory markers	IPE and inulin reduced HOMA-IR compared to control
Mueller et al. (2020) [53]	Randomized, crossover	High-fiber diets (OmniHeart)	Circulating SCFA, glucose, insulin	Fiber intake associated with increased serum SCFA and reduced insulin
Omary et al. (2025) [54]	RCT, placebo-controlled, parallel	Intrinsic chicory root fibers, 12 weeks vs. placebo	Fecal SCFA, whole-body insulin sensitivity	Increased fecal butyrate and propionate; significant improvement in insulin sensitivity
Kirschner et al. (2025) [55]	RCT, double-blind	Inulin 30 g/day, 7 days vs. placebo	Plasma SCFA kinetics	Increased plasma production of butyrate and propionate

**Table 3:** Evidence from Clinical Studies on the Role of Dietary Fiber in the Gut Microbiota–Inflammation–Glucose Metabolism Axis in T2DM.

Reference (APA)	Study Type	Participants	Intervention Duration	Type of Fiber	Main Outcomes
Zhao et al. (2018) [56]	RCT	~43 T2DM patients	Several weeks	Dietary fiber mixture targeting SCFA-producing bacteria	Improved HbA1c, increased SCFA-producing bacteria, enhanced GLP-1 secretion
Chen et al. (2023) [57]	RCT	Reported in full text	Several weeks	High-fiber diet	Improved glucose homeostasis, reduced inflammation, increased Akkermansia and Bifidobacterium
Birkeland et al. (2020) [58]	RCT	Reported in full text	8–12 weeks	Inulin-type fructans	Altered gut microbiota composition and increased SCFA production
Pedersen et al. (2016) [59]	RCT	Reported in full text	6–12 weeks	Galacto-oligosaccharides	Modulated host–microbiome interactions related to glucose metabolism
Dehghan et al. (2014) [60]	RCT	Women with T2DM	Several weeks	Oligofructose-enriched inulin	Reduced inflammatory markers and metabolic endotoxemia
Aliasgharzadeh et al. (2015) [61]	RCT	Women with T2DM	Several weeks	Resistant dextrin	Improved insulin resistance and reduced inflammation
Ojo et al. (2021) [62]	Systematic review and meta-analysis	>300 participants across RCTs	Variable	Various dietary fibers	Increased Bifidobacterium and reduced inflammatory markers

**Table 4:** Comparative effects of soluble and insoluble dietary fibers on glycemic control in diabetes.

Reference	Study Type	Participants	Intervention Duration	Type of Fiber	Main Outcomes
Abutair, A. S., Naser, I. A., & Hamed, A. T. (2016) [63].	Randomized controlled trial	40 patients with T2DM	8 weeks	Soluble fiber (psyllium)	Significant reductions in fasting blood glucose, HbA1c, insulin levels, and HOMA-IR
Silva, F. M., et al. (2020) [64].	Systematic review and network meta-analysis	2685 participants	Variable	Soluble dietary fibers	Soluble fibers associated with greater reductions in HbA1c and FPG
Honsek, C., et al. (2018) [65].	Randomized placebo-controlled trial	180 individuals with impaired glucose tolerance	2 years	Insoluble cereal fiber	Modest improvements in HbA1c and postprandial glucose, with stronger effects in specific subgroups
Kabisch, S., et al. (2019) [66].	Secondary analysis of RCT	136 participants	1 year	Insoluble cereal fiber	Improved glycemic parameters in participants with impaired fasting glucose

## Data Availability

Data sharing is not applicable to this article, as no new data were created or analyzed in this study.
